# Biochemical and immunological characterization of a novel monoclonal antibody against mouse leukotriene B_4_ receptor 1

**DOI:** 10.1371/journal.pone.0185133

**Published:** 2017-09-18

**Authors:** Fumiyuki Sasaki, Tomoaki Koga, Kazuko Saeki, Toshiaki Okuno, Saiko Kazuno, Tsutomu Fujimura, Yasuyuki Ohkawa, Takehiko Yokomizo

**Affiliations:** 1 Department of Biochemistry, Juntendo University School of Medicine, Tokyo, Japan; 2 Laboratory of Proteomics and Biomolecular Science Research Support Center, Juntendo University Graduate School of Medicine, Tokyo, Japan; 3 Laboratory of Bioanalytical Chemistry, Tohoku Medical and Pharmaceutical University, Miyagi, Japan; 4 Division of Transcriptomics, Medical Institute of Bioregulation, Kyushu University, Fukuoka, Japan; Chiba University Graduate School of Medicine, JAPAN

## Abstract

Leukotriene B_4_ (LTB_4_) receptor 1 (BLT1) is a G protein-coupled receptor expressed in various leukocyte subsets; however, the precise expression of mouse BLT1 (mBLT1) has not been reported because a mBLT1 monoclonal antibody (mAb) has not been available. In this study, we present the successful establishment of a hybridoma cell line (clone 7A8) that produces a high-affinity mAb for mBLT1 by direct immunization of BLT1-deficient mice with mBLT1-overexpressing cells. The specificity of clone 7A8 was confirmed using mBLT1-overexpressing cells and mouse peripheral blood leukocytes that endogenously express BLT1. Clone 7A8 did not cross-react with human BLT1 or other G protein-coupled receptors, including human chemokine (C-X-C motif) receptor 4. The 7A8 mAb binds to the second extracellular loop of mBLT1 and did not affect LTB_4_ binding or intracellular calcium mobilization by LTB_4_. The 7A8 mAb positively stained Gr-1-positive granulocytes, CD11b-positive granulocytes/monocytes, F4/80-positive monocytes, CCR2-high and CCR2-low monocyte subsets in the peripheral blood and a CD4-positive T cell subset, Th1 cells differentiated *in vitro* from naïve CD4-positive T cells. This mAb was able to detect Gr-1-positive granulocytes and monocytes in the spleens of naïve mice by immunohistochemistry. Finally, intraperitoneal administration of 7A8 mAb depleted granulocytes and monocytes in the peripheral blood. We have therefore succeeded in generating a high-affinity anti-mBLT1 mAb that is useful for analyzing mBLT1 expression *in vitro* and *in vivo*.

## Introduction

Leukotriene B_4_ receptor 1 (BLT1) is one of the class A G protein-coupled receptors (GPCRs) for leukotriene B_4_ (LTB_4_) [1, 2], which is a pro-inflammatory lipid mediator [3] derived from arachidonic acid [4, 5]. BLT1 is expressed in various leukocytes such as neutrophils [6], eosinophils [2, 7], dendritic cells (DC) [8, 9], monocytes/macrophages [10, 11], mast cells [12], osteoclasts [13], and activated/differentiated T cells [14, 15, 16]. It initiates a cascade of inflammatory responses including recruitment of leukocytes [17, 18, 19, 20, 21], phagocytosis of microbes [22, 23, 24], and production of pro-inflammatory cytokines/chemokines [25, 26, 27, 28]. Although the role of BLT1 in inflammatory responses is well documented, it has been difficult to analyze the cell-specific expression and function of mouse BLT1 (mBLT1) because there has not been a monoclonal antibody (mAb) available to detect mBLT1.

Monoclonal antibodies (mAbs) have long been used as analytical tools for the identification, localization, and quantification of their specific antigens. They also have been used as therapeutic agents for the treatment of human diseases such as rheumatoid arthritis [29, 30, 31] and Crohn’s disease [32, 33, 34]. Highly specific anti-GPCR mAbs are particularly helpful for defining the anatomical localization as well as the biochemical properties of the receptors. These mAbs are used to evaluate GPCR expression in living cells (by flow cytometry and confocal microscopy), membrane extracts (by western blotting), and fixed tissue sections (by immunohistochemistry) [35, 36, 37, 38, 39]. Specific GPCR mAbs have also be used to purify receptors [40, 41], characterize receptor dimers [42, 43, 44], identify receptor-associating protein partners (by co-immunoprecipitation) [45, 46], and stabilize GPCRs for crystallization [47]. Anti-GPCR mAbs are also valuable tools for studying the signaling and functions of orphan receptors. Approximately 80 GPCRs involved in cancer and inflammatory and metabolic disorders have been proposed as possible targets of antibody-based therapy [48]. Because mAbs do not cross the blood-brain barrier because of their high molecular weight, anti-GPCR mAbs could be used to target GPCRs expressed in the periphery. Thus, mAbs have the potential to treat various diseases without adverse effects in the brain [49].

Although specific Abs against variety of antigens, including some GPCRs, have been developed using phage display technology [50, 51, 52], the most common method of generating Abs, by immunizing animals with target protein, has been generally unsuccessful in the case of GPCRs. In fact, most of the available anti-GPCR Abs are polyclonal Abs purified from the serum of animals immunized with synthetic peptides corresponding to amino acid sequences within the amino (extracellular)-terminal and carboxyl (intracellular)-terminal domains and the extra- and intra-cellular loops of the GPCRs. However, commercially available polyclonal Abs often show non-specific reactivity and cross-reactivity with other plasma membrane proteins, making it difficult to clearly distinguish the specific Ab-GPCR binding from non-specific binding.

In the present study, we report the establishment of a highly specific and highly sensitive mAb for endogenous and overexpressed mBLT1 generated by immunization with mBLT1-overexpressing cells.

## Materials and methods

### Materials

Alexa Fluor (AF) 488-conjugated anti-mouse Immunoglobulin G Ab (anti-mIgG-AF488) was purchased from Thermo Fisher Scientific (Waltham, MA). R-phycoerythrin (PE)-conjugated anti-mIgG and anti-rat IgG (rIgG) Abs were purchased from Beckman Coulter (Brea, CA). Unless otherwise noted, all Abs were purchased from eBioscience/Thermo Fisher Scientific.

### Mice

BLT1-deficient (BLT1-KO: *Ltb4r1*^-/-^) mice were generated as described previously [53, 54] and backcrossed with BALB/c or C57BL/6 mice for more than 12 generations. Wild-type (WT) mice (BALB/c) were purchased from Japan SLC (Shizuoka, Japan) or Kyudo (Saga, Japan). All mice were maintained in a filtered-air laminar-flow enclosure in a specific pathogen-free facility and given standard laboratory food and water. All mice were anesthetized by intraperitoneal (i.p.) injection of ketamine (100 mg/kg) and xylazine (10 mg/kg).

### Ethics statement

All animal experiments were approved by the Ethical Committee for Animal Experiments in Kyushu University and Juntendo University. All the studies in this manuscript were carried out in accordance with approved guidelines and regulations.

### Plasmids

The pCXN2 vector [55] was used for the expression of various GPCRs. Constructs encoding various untagged or N-terminally FLAG-tagged GPCRs were constructed in-house as previously described [56].

### Cell culture

L1.2 cells (a murine pre-B lymphoma cell line; kindly gifted by Dr. Eugene C. Butcher, Stanford University) were cultured in RPMI 1640 (Wako) supplemented with 10% fetal calf serum (FCS) (Thermo Fisher Scientific), 2 mM L-glutamate, 1 mM sodium pyruvate, 50 μM 2-mercaptoethanol, 100 U/ml penicillin, and 100 μg/ml streptomycin (P/S). CHO cells (a Chinese hamster ovary cell line; ATCC) were cultured in Ham’s F12 (Wako) supplemented with 10% FCS and P/S. SP2/0-Ag14 cells (a murine myeloma cell line; ATCC) were cultured in DMEM (Wako) supplemented with 10% FCS and P/S. Anti-mBLT1 mAb-producing hybridoma cells were maintained in Hybridoma-SFM (Thermo Fisher Scientific) supplemented with 10% FCS, 5% BM-Condimed (Sigma-Aldrich, St. Louis, MO), 2 mM L-glutamine, and P/S. Anti-Gr-1 (Ly6G/Ly6C) mAb-producing hybridoma cells (RB6-8C5; obtained from Cell Resource Center for Biomedical Research, Tohoku University) and anti-FLAG mAb-producing hybridoma cells (2H8; in-house) [56] were maintained in RPMI 1640 supplemented with 10% FCS and P/S.

### Transfection

L1.2 and CHO cells were transfected with expression vectors for various GPCRs using Lipofectamine LTX and PLUS reagent (Thermo Fisher Scientific) according to the manufacturer’s protocol [56].

### Establishment of an anti-mouse BLT1 mAb

To establish an anti-mBLT1 mAb, BLT1-WT (*Ltb4r1*^+/+^) and BLT1-KO mice (BALB/c, male and female, 8–10 weeks old) were immunized with L1.2-mBLT1 cells. I.p. injections of 1–5 × 10^7^ intact cells were administered once a week for 8 weeks, followed by four weekly immunizations of the same cells together with an equal volume of monophosphoryl lipid A/trehalose dicorynomycolate (MPL/TDM) adjuvant (Sigma-Aldrich). Plasma was collected 3 days after the 1st–8th immunizations. Anti-mBLT1 Ab titers were measured by flow cytometry using mBLT1-overexpressing CHO (CHO-mBLT1) cells and anti-mIgG-AF488. Three days after the final immunization, splenocytes and popliteal lymph node cells were collected and fused with SP2/0-Ag14 cells at a ratio of 10:1 using 50% (w/v) polyethylene glycerol (Sigma-Aldrich). Cells were resuspended in HAT medium (Hybridoma-SFM medium containing 10% FCS, 5% BM-condimed, 2 mM L-glutamine, 0.1 mM hypoxanthine, 16 μM thymidine, 0.73 μM aminopterin, and P/S) and seeded into 96-well plates. After confirming colony formation, culture supernatant was screened by flow cytometry using CHO-mBLT1 cells and anti-mIgG-AF488. Screening of approximately 1,200 clones identified a positive clone producing the anti-mBLT1 Ab. The monoclonal hybridoma cells were established by limiting dilution, and designated 7A8.

### Antibody purification and labeling

Abs were affinity-purified from the culture supernatant using Protein G Sepharose (GE Healthcare) according to the manufacturer’s protocol. Affinity-purified mAbs were separated by SDS-PAGE on 10% acrylamide gels and stained with Coomassie brilliant blue. The concentration of purified Ig was determined by UV absorbance at 280 nm. The isotype of the 7A8 mAb was determined using a mouse mAb isotype kit (Hycult biotechnology). Biotinylated 7A8 mAb (7A8-Biotin) or AF488-labeled mAb (7A8-AF488) were prepared using sulfosuccinimidobiotin (Thermo Fisher Scientific) or AF488 carboxylic acid, tetrafluorophenyl ester, bis (triethylammonium salt) (Thermo Fisher Scientific) according to the manufacturer’s protocol, respectively.

### Flow cytometry

CHO-mBLT1 cells and mock transfectants were incubated with 10-fold-diluted mouse plasma or 2-fold-diluted hybridoma supernatant in PBS/EDTA [phosphate-buffered saline (PBS) with 2 mM disodium ethylenediaminetetraacetic acid (EDTA) (pH 7.4)] containing 2% FCS for 30 min at 4°C in 96-well V-bottom plates. After washing with PBS/EDTA, cells were stained with 10 μg/ml anti-mIgG-AF488 for 30 min. To detect overexpressed BLT1 and other GPCRs, transfected CHO cells were incubated with 7A8, anti-human BLT1 (hBLT1) (14F11; Becton Dickinson, Franklin Lakes, NJ), anti-human chemokine (C-X-C motif) receptor 4 (hCXCR4) (12G5; Becton Dickinson), or anti-FLAG (2H8; in-house) and then labeled with 1 μg/ml PE-conjugated anti-mIgG or anti-rIgG Abs. For staining of endogenous BLT1, peripheral blood was collected from BLT1-WT and BLT1-KO mice (BALB/c, male, 8–12 weeks old), or WT mice (BALB/c, male, 8–12 weeks old) using heparinized syringe (Novo heparin; Mochida Pharmaceutical, Tokyo, Japan) and peripheral blood leukocytes (PBL) were isolated by sedimentation of red blood cells using 2% dextran 500 (Sigma-Aldrich). After red blood cell lysis with hemolysis buffer (150 mM NH_4_Cl, 10 mM NaHCO_3_, and 0.1 mM EDTA-Na_2_), PBL were incubated with 5 μg/ml anti-CD16/32 mAb (Biolegend, San Diego, CA) to block Fc receptors and stained with 5 μg/ml 7A8-Biotin with 1.25 μg/ml anti-Gr-1-fluorescein isothiocyanate (FITC) (RB6-8C5), 1.25 μg/ml anti-CD11b-allophycocyanin (APC) (M1/70), 5 μg/ml anti-F4/80-PerCP-Cy5.5 (BM8), 2.5 μg/ml anti-CD4-FITC (RM4-5), 0.5 μg/ml anti-CD8alpha-FITC (53–6.7), 5 μg/ml anti-B220-FITC (RA3-6B2; Becton Dickinson), or 2.5 μg/ml anti-mouse chemokine (C-C motif) receptor 2 (CCR2)-PE (#475301; R&D Systems, Minneapolis, MN) mAbs. Then cells were labeled with 1 μg/ml streptavidin (SA)-PE (Thermo Fisher Scientific) or 1 μg/ml SA-FITC (Thermo Fisher Scientific). Splenocytes and lymph node cells were collected from WT mice (C57BL/6J, male, 7–8 weeks). After hemolysis and washing, cells were stained with 2 μg/ml biotinylated anti-mouse TER-119 (TER-119), CD49b (DX5), CD8alpha (53–6.7), CD11b (M1/70), CD11c (N418), B220 (RA3-6B2), and CD25 (PC61.5) mAbs for 30 min, followed by incubation with SA-MicroBeads (Miltenyi Biotech, Bergisch Gladbach, Germany). The naïve CD4^+^T cells were purified by negative selection using AutoMACS (Miltenyi Biotech) and were then suspended with RPMI1640 medium containing 1 μg/ml anti-CD28 (37.51) mAb, 10% FCS, 50 μM 2-mercaptoethanol, and P/S. Cells were transferred into 24 well plates coated with anti-CD3 (145-2C11) mAb and *in vitro* differentiated into CD4^+^ T helper cell subsets, type 0 (Th0), type 1 (Th1), and type 2 (Th2) cells in the presence with 10 μg/ml anti-IFNgamma (R4-6A2) and 10 μg/ml anti-IL-4 (11B11) mAbs (Th0), 100 ng/ml Murine IL-12 p70 (Peprotech, Rocky Hill, NJ) and 10 μg/ml anti-IL-4 mAb (Th1), and 50 ng/ml Murine IL-4 (Peprotech) and 10 μg/ml anti-IFNgamma mAb (Th2) for 7 days. Cells were stained with 5 μg/ml 7A8-AF488 or mIgG-AF488. Dead cells were excluded with 7-amino-actinomycin D (7AAD; Becton Dickinson). Cells were analyzed on a FACSCalibur flow cytometer (Becton Dickinson).

### Immunofluorescence staining

CHO-mBLT1 cells and mock transfectants were seeded on glass-bottom dishes (Matsunami Glass, Osaka, Japan) coated with collagen (Cellmatrix Type I-P; Nitta Gelatin, Osaka, Japan). After 48 hr, cells were fixed with 4% paraformaldehyde (PFA) in PBS containing 10 mM glycine (PBS-G) for 5 min, washed with PBS-G, blocked with 3% bovine serum albumin (BSA) in PBS for 10 min, and stained with 10 μg/ml 7A8 mAb in PBS containing 1% BSA for 30 min followed by 10 μg/ml anti-mIgG-AF488. After washing with PBS, slides were mounted with Mowiol mounting medium containing 2.5% 1,4-diazobicyclo-[2.2.2]-octane and observed by confocal microscopy using a LSM510 instrument (Carl Zeiss, Oberkochen, Germany). Spleens were removed form BLT1-WT and BLT1-KO mice (C57BL/6, male, 20–24 weeks old), fixed with 4% PFA for 30 min, soaked with 10% and 30% sucrose in PBS for over 2 hr, embedded in O.C.T. compound (Sakura Finetek Japan, Tokyo, Japan), and sliced at a 20 μm thickness using a cryostat. Frozen sections were fixed with 4% PFA/PBS and blocked with 5% BSA and 0.5% Triton X-100 in PBS for 1 hr, then stained with 10 μg/ml 7A8 and AF647-conjugated anti-Gr-1 mAb. After washing with 0.1% Tween 20 in PBS, sections were stained with a 1:500 dilution of horseradish peroxidase-conjugated anti-mIgG Ab (Rockland Immunochemicals, Limerick, PA), followed by staining with AF488-labeled tyramide (Thermo Fisher Scientific). Nuclei were stained with 1 mg/ml DAPI (Sigma-Aldrich). Sections were observed using a TCS SP8 confocal microscope (Leica Microsystems, Wetzlar, Germany).

### Determination of the epitope recognized by 7A8

Four peptides (mBLT1_1-21_: MAANTTSPAAPSSPGGMSLSL, mBLT1_76-94_: FLHFLARGTWSFREMGCRL, mBLT1_159-190_: TVKWNNRTLICAPNYPNKEHKVFHLLFEAITG, and mBLT1_240-271_: LVNLVEAGRTVAGWDKNSPAGQRLRLARYVLI) were synthesized using a PSSM-8 peptide synthesizer (Shimadzu, Kyoto, Japan). The 7A8 mAb (1 μg/ml) was pre-incubated with 0.005–100 μM of each of the peptides for 30 min at 37°C. L1.2-mBLT1 cells and mock transfectants were incubated with each Ab-peptide mixture for 30 min at 4°C. After washing with PBS/EDTA, cells were stained with 5 μg/ml anti-mIgG-AF488 and analyzed on a flow cytometer.

### Surface plasmon resonance (SPR)

The 7A8 mAb was pre-concentrated with 10 mM acetate (pH 5.0) and immobilized on a CM5 sensor chip (GE Healthcare, Chicago, IL) with HBS-EP buffer [0.01 M HEPES (pH 7.4), 0.15 M NaCl, 3 mM EDTA, and 0.05% Surfactant P20] that had been activated with 1-ethyl-3-(3-dimethylaminopropyl)-carbodiimide and N-hydroxysuccinimide and blocked with ethanolamine-HCl (pH 8.5). The four peptides (10 μM) were injected individually at a flow rate of 30 μl/min for 2 min. Resonance units (RU) were measured using a Biacore T-200 SPR spectrometer (GE Healthcare).

### Ligand binding assay

Microsomal fractions (5 μg) of mBLT1-overexpressing CHO and L1.2 cells or mock transfectants were prepared as described previously [57]. Proteins were incubated with 0.5 nM [^3^H] labeled LTB_4_ ([^3^H]LTB_4_) in the presence or absence of 10 μg/ml 7A8 mAb for 1 hr. A control sample for non-specific binding was prepared with the [^3^H]LTB_4_-mAb mixture and 1 μM unlabeled LTB_4_. Samples were transferred onto a GF/C filter and washed with binding buffer [50 mM Tris-HCl (pH 7.5), 10 mM MgCl_2_, and 10 mM NaCl]. Dried filters were immersed in MicroScint-O scintillation fluid (PerkinElmer, Waltham, MA), and the radioactivity was measured using a TopCount scintillation counter (PerkinElmer).

### Ligand-induced calcium mobilization assay

Briefly, L1.2-mBLT1 cells, L1.2-FLAG-mBLT1 cells, or mock transfectants were pre-incubated with 7A8 (1 and 10 μg) or control mIgG_1_ (10 μg) and then loaded with Fluo 3-acetxymethyl (Dojindo Laboratories, Kumamoto, Japan) in a Hank’s balanced salt solution (HBSS)-based loading buffer containing 20 mM 2-[4-(2-hydroxyethyl)-1-piperazinyl]ethanesulfonic acid (pH 7.4), 2.5 mM probenecid (Sigma-Aldrich), and 0.04% Pluronic F-127 (Thermo Fisher Scientific). Cells were then stimulated with 0.01–1000 nM LTB_4_. Assay plates were analyzed using a FlexStation 3 (Molecular Devices, Sunnyvale, CA).

### Depletion assay

WT mice (BALB/c, female, 8–10 weeks old) were i.p. injected with 100 μg of 7A8, 1A8 (anti-Ly6G mAb; Bio X Cell, West Lebanon, NH), or anti-Gr-1 mAbs, or PBS (as a control). PBL were collected at 1 day after mAb injection as described above (see “Flow cytometry”), and were then stained with mAbs and analyzed by flow cytometry.

### Statistics

Data are expressed as the mean ± SEM. ANOVA tests were used for multiple comparisons. *P*-values <0.05 were considered statistically significant. All statistical analyses were performed using Prism, version 5.0 (GraphPad Software, La Jolla, CA).

## Results and discussion

### Establishment of a hybridoma cell line that produces an anti-mouse BLT1 monoclonal antibody

**Fig 1:**
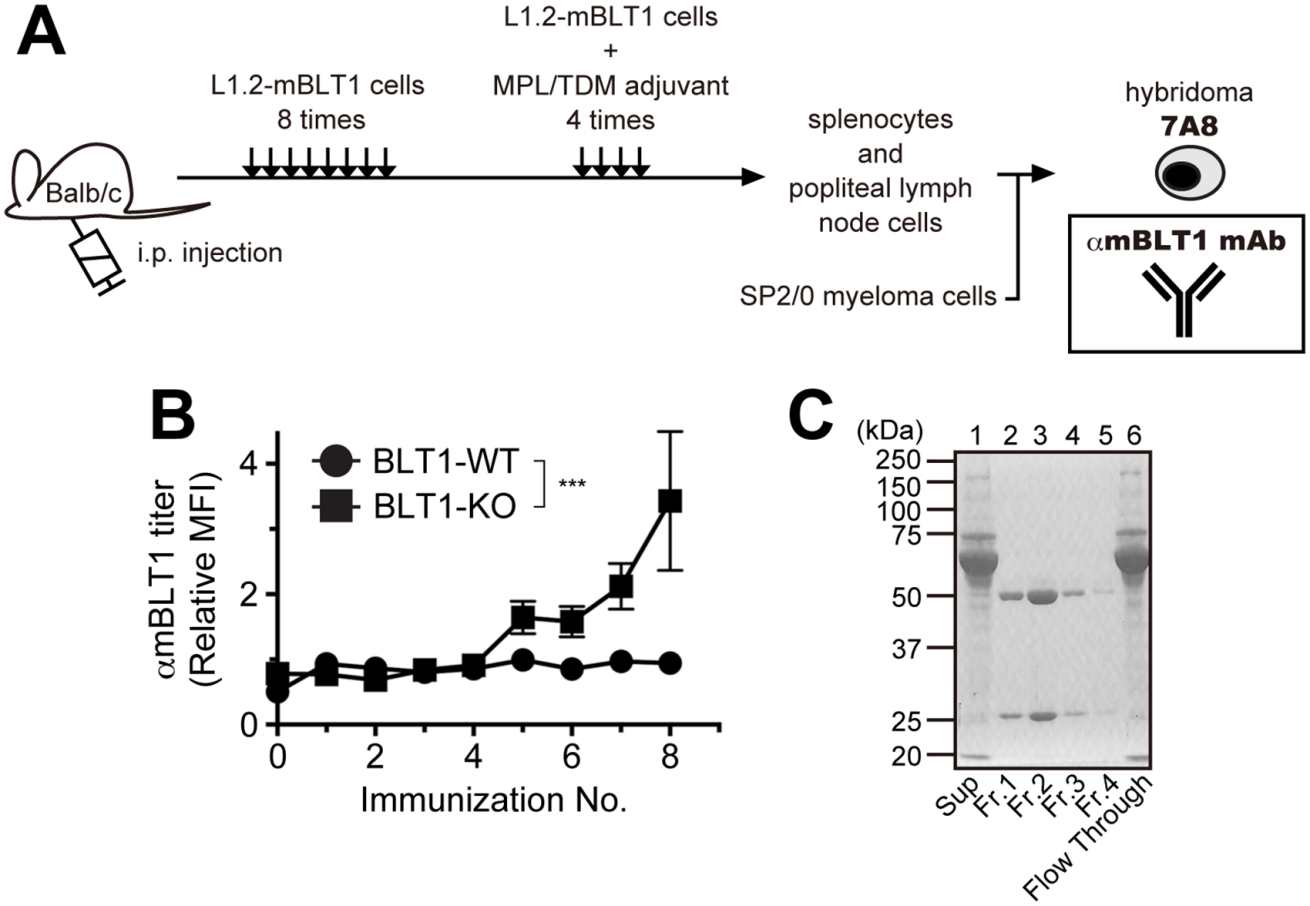
Establishment of a novel anti-mBLT1 mAb. **(A)** The strategy for generation of anti-mBLT1 mAbs by immunization of BLT1-KO mice (BALB/c) with intact L1.2-mBLT1 cells. **(B)** Ab titers in the plasma of BLT1-WT and BLT1-KO mice after immunization with L1.2-mBLT1 cells (n = 6 for BLT1-WT mice, n = 8 for BLT1-KO mice). The mean fluorescent intensity (MFI) was determined by flow cytometry. The relative MFI was defined as the MFI of binding to CHO-mBLT1 cells divided by the MFI of binding to mock-transfected cells. Data were analyzed by two-way ANOVA. ***, *p* < 0.001. **(C)** SDS-PAGE analysis of 7A8 mAb purified from hybridoma supernatant using Protein G Sepharose.

To establish a hybridoma that produces a high-affinity mAb for mBLT1, BLT1-WT and BLT1-KO mice were immunized with L1.2-mBLT1 cells that stably expressed untagged mBLT1 (Fig 1A). After eight i.p. injections of L1.2-mBLT1 cells, mice received four additional immunizations with the same cells plus MPL/TDM adjuvant. Plasma from BLT1-KO mice, but not BLT1-WT mice, was found to be immunoreactive to BTL1 (Fig 1B). Approximately 1,200 hybridoma clones were generated by fusing SP2/0 myeloma cells with splenocytes and lymph node cells from immunized BLT1-KO mice. Culture supernatant from one clone, 7A8, positively stained CHO-mBLT1 cells by flow cytometry. Immunoglobulin was purified from the 7A8 culture supernatant using Protein G Sepharose, and the purity and recovery of 7A8 mAb was analyzed by SDS-PAGE (Fig 1C). The isotype of the 7A8 mAb was determined to be IgG_1_. The average yield of functional 7A8 mAb was approximately 2.3 mg from 100 ml of culture supernatant.

### 7A8 mAb specifically recognizes mouse BLT1

**Fig 2:**
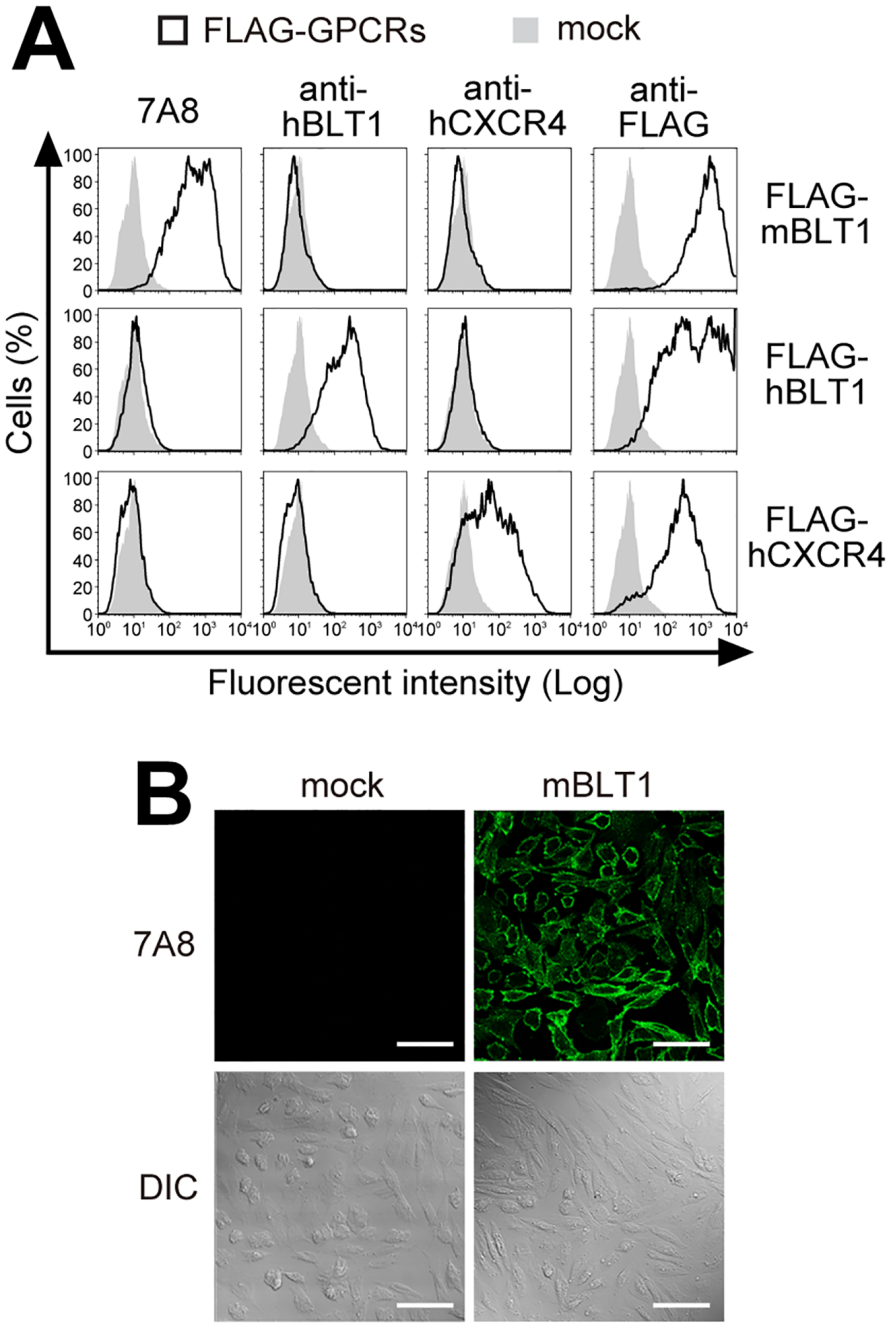
The 7A8 mAb detects mBLT1-overexpressing cells. **(A)** FLAG-mBLT1, FLAG-hBLT1, and FLAG-hCXCR4-overexpressing CHO cells (black outlines) and mock transfectants (gray filled histograms) were stained with anti-mBLT1 (7A8), anti-hBLT1, anti-hCXCR4, and anti-FLAG (2H8) mAbs. **(B)** Immunofluorescence staining of CHO-mBLT1 cells (right) and mock transfectants (left) using 7A8. DIC: differential interference contrast. Scale bars: 50 μm.

To confirm the specificity of the 7A8 mAb, mock-transfected cells and cells transfected with FLAG-tagged mBLT1 (FLAG-mBLT1), hBLT1 (FLAG-hBLT1), or hCXCR4 (FLAG-hCXCR4) were stained with purified 7A8 mAb (Fig 2A). Staining with the 7A8 mAb resulted in a 1.5-log shift in fluorescence for FLAG-mBLT1 cells, but not for FLAG-hBLT1 or FLAG-hCXCR4 cells. The 7A8 mAb did not cross-react with other GPCRs, including mouse leukotriene B_4_ receptor 2 (BLT2) and chemokine (C-C motif) receptor 7 (CCR7), human sphingosine-1-phosphate receptor 1 (S1P1) and cysteinyl leukotriene receptor 1 (CysLT1), and guinea pig platelet-activating factor receptor (PAFR) (data not shown). By immunofluorescence, 7A8 strongly stained CHO-mBLT1 cells but not mock transfectants (Fig 2B). These data demonstrate that 7A8 is a specific mAb for mBLT1.

### The 7A8 mAb recognizes the second extracellular loop of mBLT1

**Fig 3:**
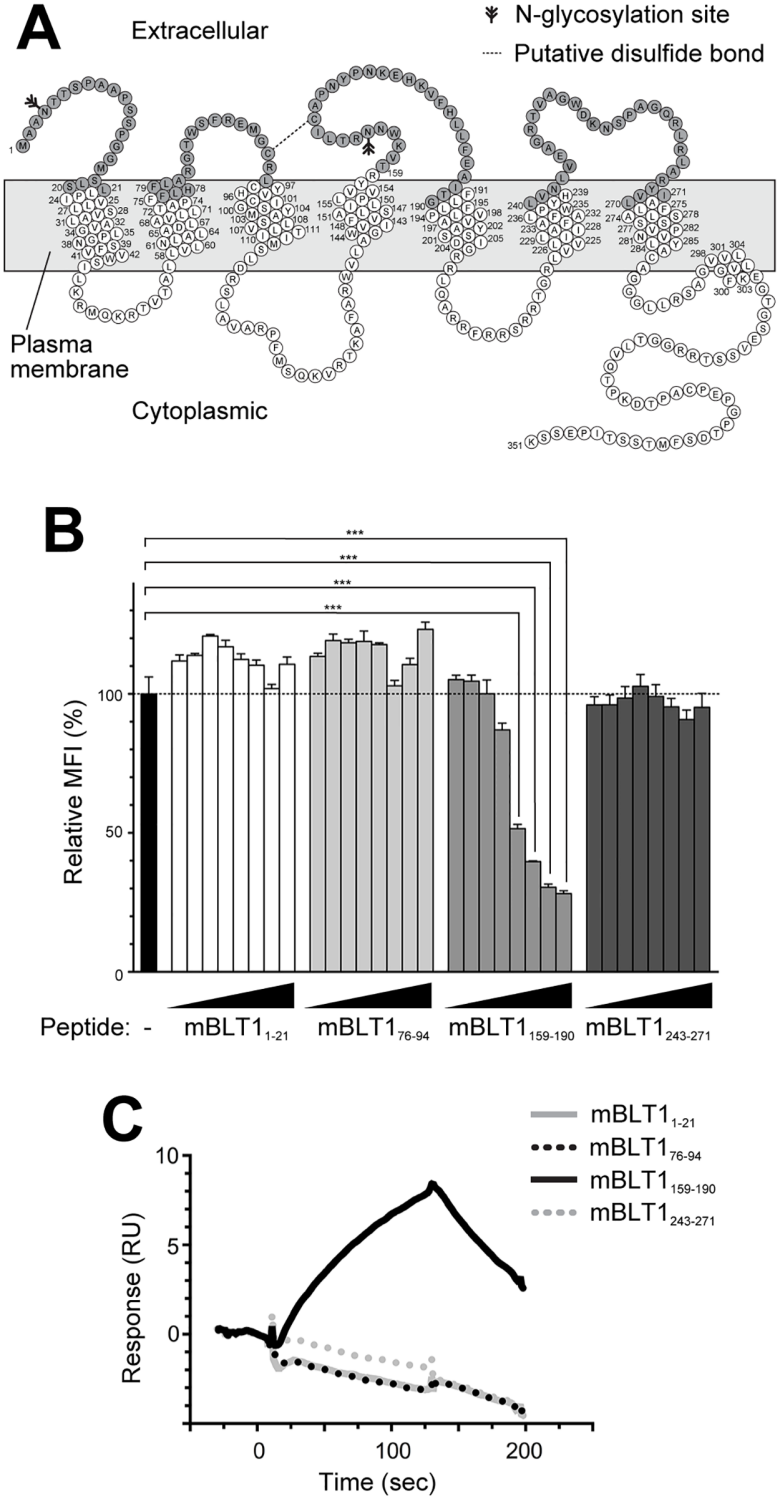
Determination of the epitope recognized by 7A8 mAb by flow cytometry and SPR. **(A)** A secondary structure diagram of the expected topology of mBLT1. The extracellular domains of mBLT1 are indicated in gray. **(B)** Flow cytometry-based competition assay with 7A8 mAb and synthetic peptides corresponding to the extracellular domains of mBLT1 (mBLT1_1-21_, mBLT1_76-94_, mBLT1_159-190_, and mBLT1_240-271_). The four peptides (0.005–100 μM) were pre-incubated with 7A8 mAb (1 μg/ml) and then used to stain CHO-mBLT1 cells and mock transfectants. Data were analyzed by one-way ANOVA, followed by the Bonferroni *post-hoc* test: ***, *p* < 0.001. **(C)** Affinity and kinetic measurements for 7A8 mAb by Biacore analysis. The four peptides were flowed over 7A8 immobilized onto a sensor chip, and the binding interactions were measured in resonance units (RU).

To determine the epitope of mBLT1 recognized by 7A8, four peptides with sequences derived from the extracellular domains of mBLT1 (an N-terminal region and three harboring loops) (Fig 3A) were synthesized and used in a competition assay with 7A8. As shown in Fig 3B, the binding reactivity of 7A8 was clearly decreased in the presence of a peptide comprising amino acids 159–190 of mBLT1 (mBLT1_159-190_), which is located in the second extracellular loop. Next, the binding of 7A8 to mBLT1_159-190_ was directly examined by SPR (Fig 3C). The 7A8 mAb bound directly to mBLT1_159-190_ with an association (*k*_*a*_) and dissociation rate constant (*k*_*d*_) of 1.05 × 10^4^ M^-1^ s^-1^ and 6.49 × 10^−3^ s^-1^, respectively. The equilibrium dissociation constant (*K*_*D*_) was 6.16 × 10^−7^ M. However, the 7A8 mAb did not inhibit the binding of LTB_4_ to mBLT1 (S1A Fig) or LTB_4_-dependent calcium mobilization through mBLT1 (S1B Fig). These data show that 7A8 binds to the liner sequence of amino acids in the second extracellular loop of mBLT1 without affecting ligand binding.

### Detection of endogenous mouse BLT1 with 7A8

**Fig 4:**
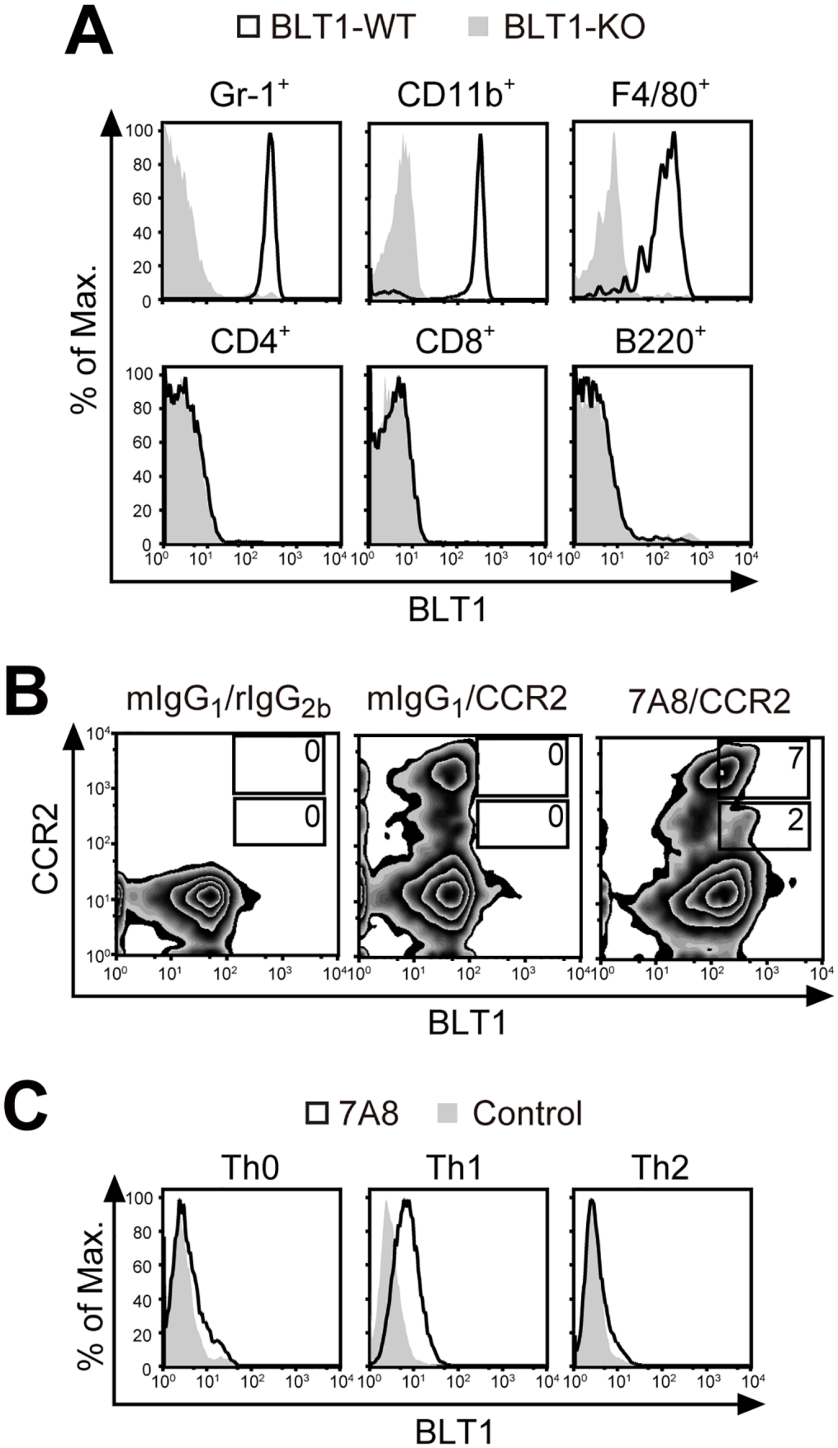
Staining of mouse peripheral blood leukocytes and T cell subsets with 7A8. Flow cytometric analysis of mouse PBL **(A, B)** and *in vitro* differentiated CD4^+^ T cell subsets **(C)** with 7A8. **(A)** PBL were stained with 7A8-Biotin and several lineage markers, and staining was analyzed separately in Gr-1^+^ granulocytes, CD11b^+^ granulocytes/monocytes, F4/80^+^ monocytes, CD4^+^ T cells, CD8^+^ T cells, and B220^+^ B cells from naïve BLT1-WT (black outlines) and BLT1-KO (gray filled histograms) mice. **(B)** Monocytes were stained with 7A8-Biotin and anti-CCR2 mAb from the peripheral blood of naïve WT mice. The mIgG_1_-Biotin and rIgG_2b_ mAb were used as isotype controls. **(C)** Th0, Th1 and Th2 cells were stained with 7A8-AF488 (black outlines) or mIgG-AF488 (gray filled histograms) as a control.

The ability of the 7A8 mAb to detect endogenous mBLT1 was analyzed next. 7A8-Biotin was prepared and used for flow cytometry. As shown in Fig 4A, 7A8-Biotin positively stained Gr-1-positive (Gr-1^+^) granulocytes, CD11b^+^ granulocytes/monocytes, and F4/80^+^ monocytes in the peripheral blood from naïve BLT1-WT mice. No 7A8 staining of naïve CD4^+^ T cells, CD8^+^ T cells, and B220^+^ B cells were observed, consistent with the previously reported lack of BLT1 expression on these cells [16]. Binding of 7A8 to Gr-1^+^ granulocytes was not observed in BLT1-KO mice but was not affected by BLT2 deficiency (data not shown), confirming the specificity of the 7A8 mAb. Both CD115^+^ (a canonical monocyte marker) and Ly6C^+^ (a classical or pro-inflammatory monocyte marker) monocytes expressed BLT1 (data not shown). Further analysis showed that BLT1 was expressed in both CCR2-high (CCR2^hi^) inflammatory and CCR2-low (CCR2^lo^) resident monocytes (Fig 4B). Furthermore, BLT1 expression was clearly detected only in a subset of CD4^+^ T cells, Th1 cells differentiated *in vitro*, but was not obvious in Th0 and Th2 cells (Fig 4C). Taken together, these data indicate that 7A8 is sensitive and specific enough to detect endogenous mBLT1.

### Validation of 7A8 for immunohistochemistry of mBLT1

**Fig 5:**
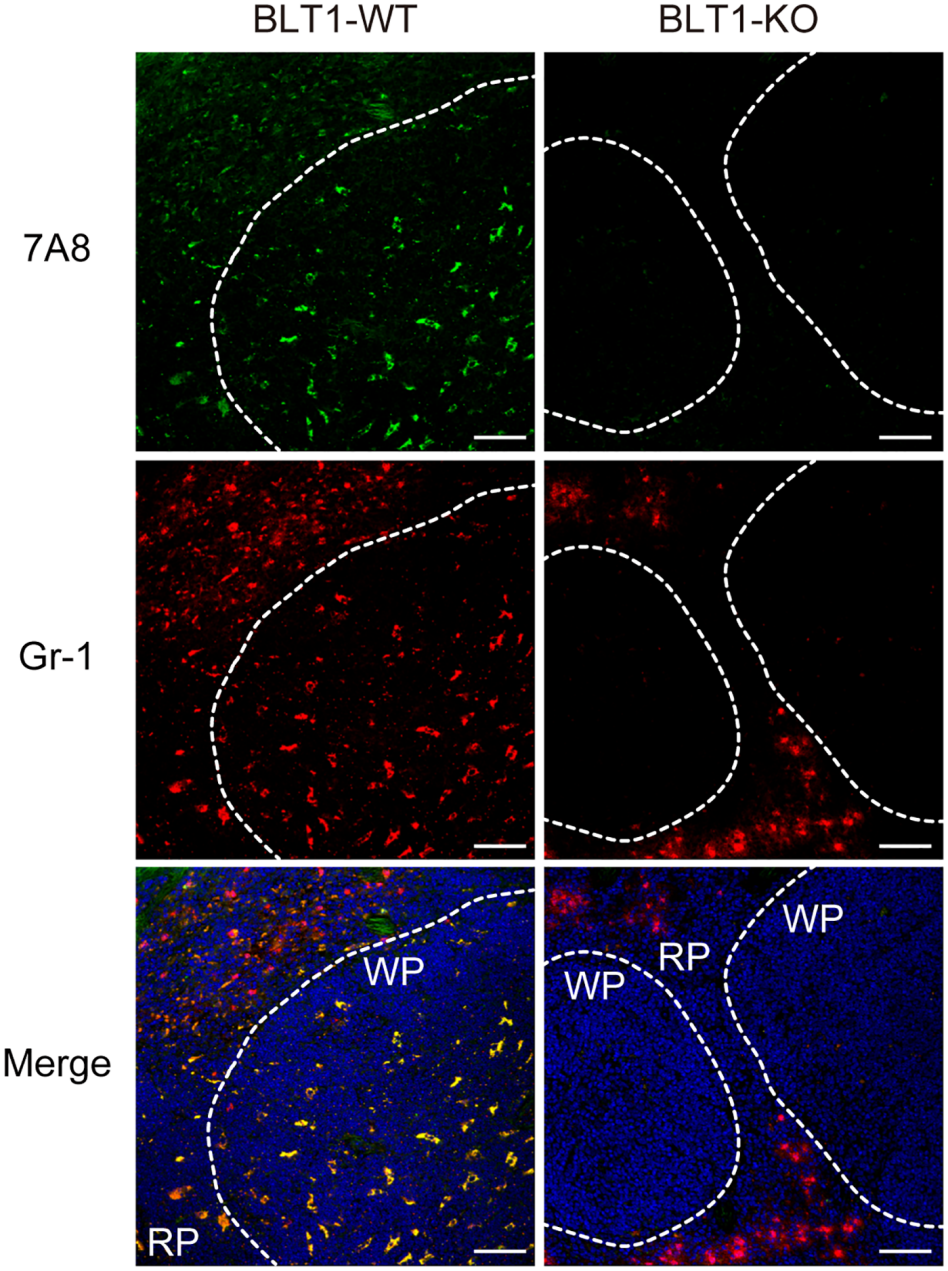
BLT1 is endogenously expressed in Gr-1-positive splenocytes of naïve mice. Spleen sections from naïve BLT1-WT (left panels) and BLT1-KO (right panels) mice were stained with 7A8 (green) and anti-Gr-1 (red) mAbs. Nuclei were visualized with DAPI (blue). RP: red pulp. WP: white pulp. Scale bars: 50 μm. Magnification: ×40.

In general, it is difficult to stain mouse tissues using monoclonal or polyclonal Abs derived from mouse due to high background staining caused by endogenous mouse IgG and mouse Fc receptors expressed on immune cells such as B cells, macrophages, and DC. We therefore tried to detect mBLT1-expressing cells in the mouse spleen with 7A8 using a tyramide signal amplification system. The 7A8 mAb was able to detect Gr-1^+^ granulocytes in the red pulp (RP) and Gr-1^+^ monocytic cells (such as monocytes, macrophages, or DC) in the white pulp (WP) of BLT1-WT mice, but 7A8 staining was not observed in BLT1-KO mice (Fig 5). These data clearly show that 7A8 mAb is a highly sensitive and specific mAb for mBLT1.

### Clone 7A8 can deplete granulocytes and monocytes from the peripheral blood

**Fig 6:**
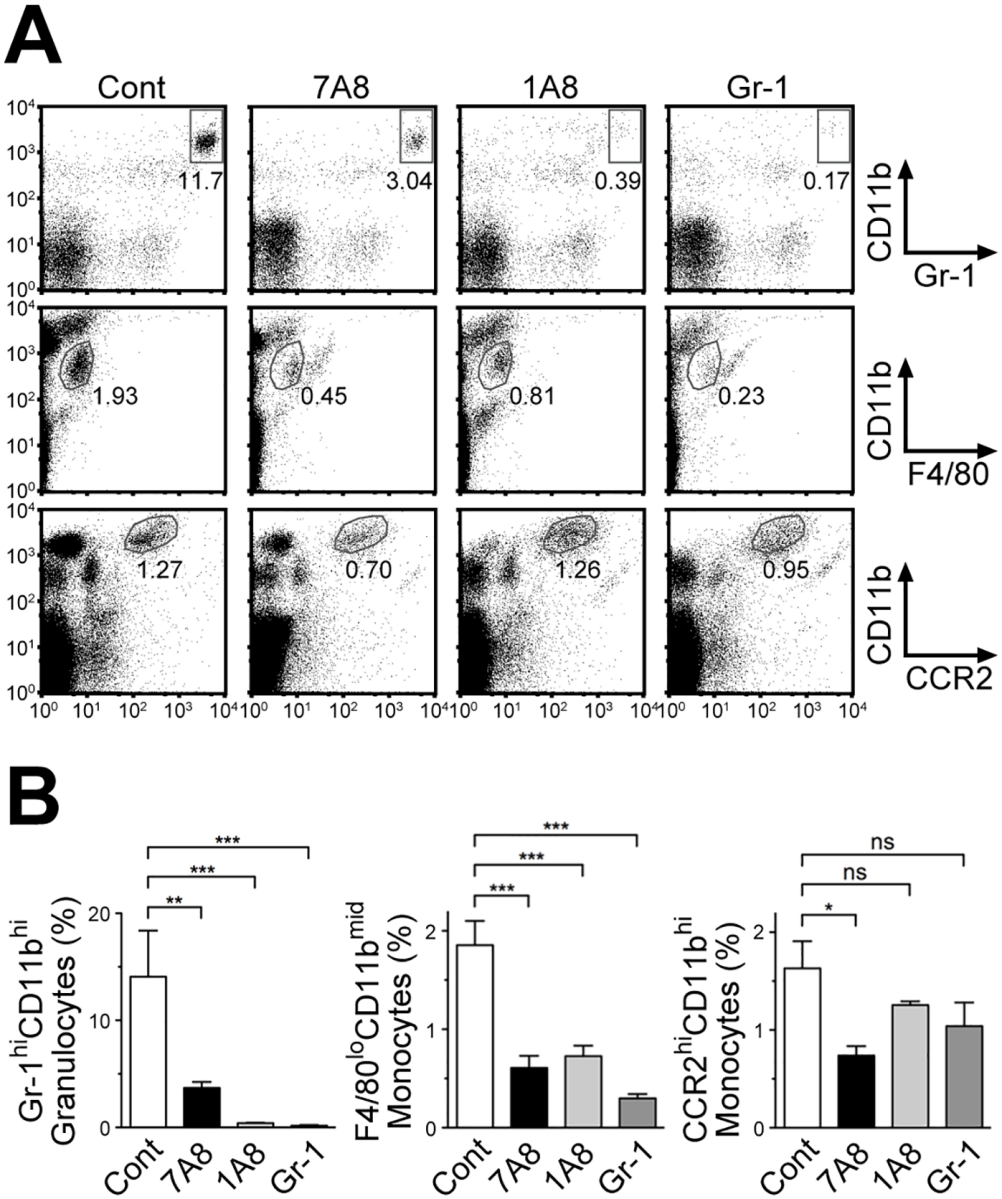
The 7A8 mAb depletes granulocytes and monocytes *in vivo*. **(A, B)** WT mice were injected intraperitoneally with 7A8, 1A8, anti-Gr-1 mAbs, or PBS as a control. **(A)** PBL were collected at 1 day after mAb injection and stained with 7A8, anti-Gr-1, anti-F4/80, and anti-CCR2 mAbs. The staining was analyzed separately in Gr-1 and CD11b (upper panels), F4/80 and CD11b (middle panels), or CCR2 and CD11b (lower panels). **(B)** The percentages of Gr-1^hi^CD11b^hi^(CCR2^-^F4/80^-^) granulocytes (left panel), F4/80^lo^CD11b^mid^(CCR2^lo^Gr-1^mid^) monocytes (middle panel), and CCR2^hi^CD11b^hi^(F4/80^lo^Gr-1^hi^) monocytes (right panel) within total PBL (n = 4–5) are shown. Cont: control. Data were analyzed by one-way ANOVA, followed by the Bonferroni *post-hoc* test. ns, not significant. *, *p* < 0.05; **, *p* < 0.01; ***, *p* < 0.001.

To examine whether 7A8 can be used for depletion of mBLT1-expressing cells *in vivo*, 7A8, 1A8, or anti-Gr-1 mAbs was administered to WT mice (100 μg/mice, i.p.). PBL were collected and analyzed by flow cytometry at 1 day after mAb injection. As shown in Fig 6A, Gr-1^hi^CD11b^hi^(CCR2^-^F4/80^-^) granulocytes and F4/80^lo^CD11b-middle (CD11b^mid^) (CCR2^lo^Gr-1^mid^) monocytes were depleted in peripheral blood in either cases; however, CCR2^hi^CD11b^hi^(F4/80^lo^Gr-1^hi^) inflammatory monocytes were eliminated only by injection of the 7A8 mAb. The 7A8 administration significantly depleted granulocytes and monocytes (Fig 6B). Administration of 1A8 and anti-Gr-1 mAb depleted most of the granulocytes with some depleting effects on monocyte subsets. Thus, the 7A8 mAb will be useful to analyze the physiological and pathophysiological roles of BLT1^+^ granulocytes and monocytes *in vivo*.

In this study, we successfully established an anti-mBLT1 mAb (clone 7A8) with high specificity and sensitivity for exogenous (Fig 2) and endogenous (Figs 4 and 5) mBLT1 by immunizing BLT1-KO mice with BLT1-overexpressing cells (Fig 1). Endogenous BLT1 is abundantly expressed in granulocytes, which have a very short life span *in vivo*. Therefore, WT mice develop peripheral immunological tolerance for BLT1, necessitating the use of BTL1-KO mice for Ab generation. Although several mAbs can be obtained by immunization with a general antigen (*e*.*g*., recombinant fusion protein or plasmid DNA) using gene-deficient mice [58, 59, 60, 61, 62, 63], little has been reported on anti-GPCR mAb generation by this strategy. Thus, this approach might be useful for generating highly specific and sensitive mAbs against other GPCRs and orphan GPCRs. We confirmed that 7A8 can be used for detection of mBLT1 on tissue by immunohistochemistry (Fig 5). In addition, 7A8 significantly depleted BLT1^+^ granulocytes and monocytes (Fig 6), but not lymphocytes (data not shown), from the peripheral blood. Furthermore, 7A8 bound the second extracellular loop of mBLT1 (Fig 3), and did not affect the LTB_4_ binding and intracellular signaling (S1 Fig). These results are consistent with previous studies showing that the transmembrane domains III, V and VI of BLT1 are involved in the LTB_4_ binding [64, 65, 66]. Our data suggests that 7A8 mAb will be a very useful tool to clarify the physiological and pathophysiological functions of BLT1 in various disease models by quickly eliminating BLT1-expressing cells without inducing the compensatory gene expression caused by BLT1 deficiency. Future studies will examine the effects of depleting BLT1-expressing cells on the onset and progression of several inflammatory diseases in animal models.

We also observed that BLT1 was expressed in circulating CCR2^hi^ inflammatory monocytes and CCR2^lo^ resident monocytes in the peripheral blood (Fig 4B). The physiological and pathophysiological roles of BLT1 in those subsets are unknown. In general, inflammatory (or classical) monocytes (CCR2^hi^ Ly6C^hi^ CX_3_CR1^lo^) are recruited to inflamed tissues, where they produce inflammatory cytokines (*e*.*g*., IL-1beta and TNF-alpha) in response to infection or tissue damage. They become differentiated into marcophages or DC during acute inflammation and chronic inflammation. In the steady state or during homeostatic inflammation, they become tissue-resident macrophages, which readily engulf dying or dead cells (including apoptotic or necrotic cells) and cellular debris, contributing to the maintenance of tissue homeostasis. Tissue-resident (or non-classical) monocytes (CCR2^lo^ Ly6C^lo^ CX_3_CR1^hi^) patrol locally to clear cellular debris. These cells can also produce anti-inflammatory cytokines (*e*.*g*., IL-10), although their cellular function is not fully understood [67, 68, 69]. Recent reports show that both monocyte subsets (classical and non-classical) can polarize into alternatively activated (or M2-type) macrophages [70, 71, 72], although the molecular mechanisms of this conversion remain poorly understood. Future studies in our group will investigate the functions of BLT1 in both classical and non-classical monocytes using the 7A8 mAb.

## Supporting information

S1 Fig 7A8 mAb does not affect interaction of LTB_4_ and mBLT1. **(A)** Competitive binding assay of 7A8 mAb with a radioactive ligand. Microsomal fractions were mixed with [^3^H]LTB_4_, and added with or without 7A8 (Total). A non-specific binding (NS) was determined with 2,000-fold concentration of non-labeled LTB_4_ in the same preparation (n = 2–3 for Total, n = 2 for NS). Data were analyzed by one-way ANOVA, followed by the Newman-Keuls *post-hoc* test: ns, not significant. **(B)** The effect of 7A8 on LTB_4_-BLT1 signaling. L1.2-mBLT1, L1.2-FLAG-mBLT1 cells or mock transfectants were incubated with 7A8 mAb, and calcium mobilization was measured by stimulation with LTB_4_ (n = 2–3). RFI: relative fluorescent intensity. Data were analyzed by two-way ANOVA: ns, not significant.
(TIF)
