# Supplementary figures and images for: Biochemical and immunological characterization of a novel monoclonal antibody against mouse leukotriene B4 receptor 1
Source: PLoS One. 2017 Sep 18;12(9):e0185133. doi: 10.1371/journal.pone.0185133 (PMC5602668; doi:10.1371/journal.pone.0185133)

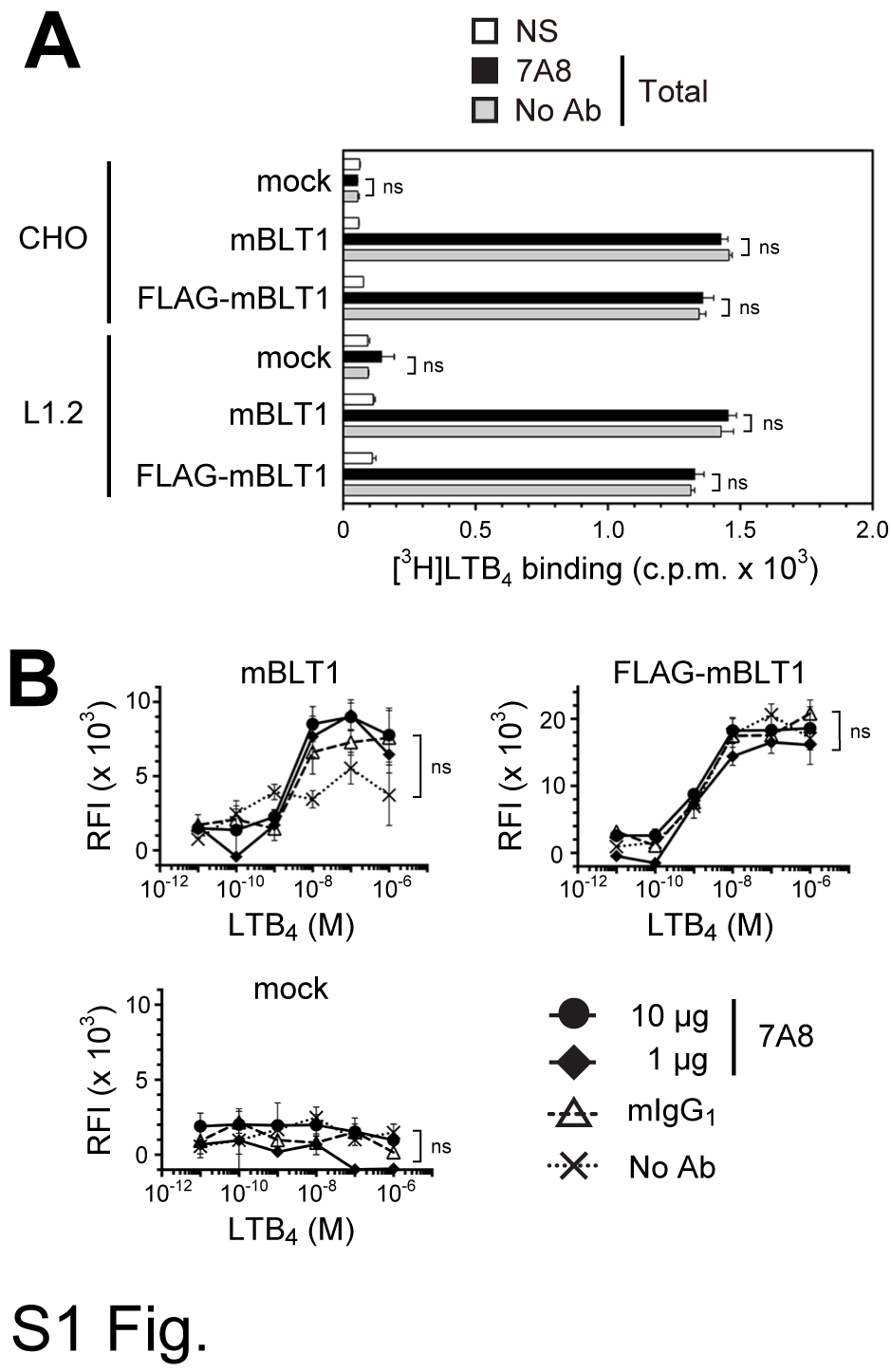

Supplement: S1 Fig — (A) Competitive binding assay of 7A8 mAb with a radioactive ligand. Microsomal fractions were mixed with [3H]LTB4, and added with or without 7A8 (Total). A non-specific binding (NS) was determined with 2,000-fold concentration of non-labeled LTB4 in the same preparation (n = 2–3 for Total, n = 2 for NS). Data were analyzed by one-way ANOVA, followed by the Newman-Keuls post-hoc test: ns, not significant. (B) The effect of 7A8 on LTB4-BLT1 signaling. L1.2-mBLT1, L1.2-FLAG-mBLT1 cells or mock transfectants were incubated with 7A8 mAb, and calcium mobilization was measured by stimulation with LTB4 (n = 2–3). RFI: relative fluorescent intensity. Data were analyzed by two-way ANOVA: ns, not significant. (TIF) [file pone.0185133.s001.tif]
